# A Novel Nogo‐A Receptor Antagonist Peptide Impedes Alzheimer's Disease Pathology by Nogo‐A/NgR/ROCK Signaling Pathway

**DOI:** 10.1002/cns.71052

**Published:** 2026-08-13

**Authors:** Zheng Zhang, Huimin Tan, Fang Shi, Jiajia Dai, Weilong Ding, Junliang Li, Yuke Wang, Rui Yang, Xinke Xu, Cheng Chen, Fei Xiao, Li‐an Huang, Xiaoyan Liu, Rui Liao, Li Yan

**Affiliations:** ^1^ Department of Pharmacy The First Affiliated Hospital of Jinan University, Jinan University Guangzhou Guangdong Province China; ^2^ Department of Neurology The First Affiliated Hospital of Jinan University Guangzhou Guangdong Province China; ^3^ Department of Neurology City Center Hospital of Zengcheng District in Guangzhou Guangzhou China; ^4^ Formula‐Pattern Research Center, School of Traditional Chinese Medicine Jinan University Guangzhou China; ^5^ Department of Pharmacology, School of Medicine Jinan University Guangzhou Guangdong Province China; ^6^ Department of Neurosurgery The First Affiliated Hospital of Jinan University Guangzhou China; ^7^ Department of Neurosurgery Guangzhou Women and Children's Medical Center Guangzhou Guangdong Province China; ^8^ School of Medicine Jinan University Guangzhou Guangdong Province China; ^9^ The First Affiliated Hospital of Guangdong Pharmaceutical University Guangzhou Guangdong Province China

**Keywords:** Alzheimer's disease, Aβ peptides, Nogo‐A receptor antagonist peptide 2(NAP2)

## Abstract

**Aims:**

To evaluate the therapeutic effects of NAP2, a novel NgR1 antagonist peptide, on Alzheimer's disease (AD) pathology and to determine whether inhibition of the Nogo‐A/NgR1/ROCK signaling axis can ameliorate neurodegenerative alterations in APP/PS1 mice.

**Methods:**

APP/PS1 transgenic mice received a three‐month NAP2 intervention. Cognitive performance was assessed using standard learning and memory tests. Amyloid‐β plaque burden, dendritic spine density, tau phosphorylation, and Aβ42 levels were examined by histological and biochemical analyses. Mitochondrial function and downstream ROCK signaling activity were also evaluated.

**Results:**

NAP2 treatment significantly improved cognitive performance in APP/PS1 mice. NAP2 reduced amyloid‐β plaque deposition, decreased Aβ42 expression, and increased hippocampal dendritic spine density. Tau hyperphosphorylation was notably attenuated. In addition, NAP2 alleviated Aβ42‐induced mitochondrial dysfunction. Mechanistic studies revealed that NAP2 interfered with ROCK signaling downstream of Nogo‐A.

**Conclusion:**

NAP2 ameliorates multiple AD‐related pathological features, an effect accompanied by the inhibition of the Nogo‐A/NgR1/ROCK pathway. These findings highlight the therapeutic potential of NAP2 to enhance resilience against Alzheimer's disease pathology.

## Graphical abstract

NAP2, a NgR1 antagonist peptide, improved cognition in APP/PS1 mice, reduced Aβ plaques and tau hyperphosphorylation, increased dendritic spine density, and alleviated mitochondrial damage, acting through inhibition of the Nogo‐A/NgR/ROCK pathway, highlighting its therapeutic potential for Alzheimer's disease.

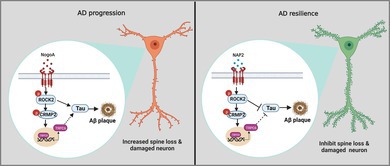

## 1 Introduction

The most common form of late‐onset dementia is Alzheimer's disease, which typically starts with a lengthy preclinical phase characterized by the progressive accumulation of amyloid‐beta plaques and tau aggregates. This preclinical period eventually transitions to a symptomatic phase marked by cognitive decline [1, 2]. The transition from the preclinical phase to the symptomatic phase of AD is still not well understood, but it is believed to coincide with changes in myelin structure and function, innate immune responses, and metabolism [3, 4]. Myelin damage refers to the destruction or impairment of the myelin sheath, a protective covering that surrounds nerve fibers in the central nervous system (CNS). This damage disrupts the normal functioning of nerve cells and can lead to various neurological symptoms. A recent study suggested that myelin dysfunction promotes amyloid‐beta plaque formation in models of AD [5, 6, 7]. Aβ and tau proteins can also interact with myelin‐associated proteins, disrupting the formation and maintenance of myelin, suggesting that myelin damage responses underlie cognitive decline [8, 9].

Nogo‐A mainly localizes to the plasma membrane of oligodendrocytes and the endoplasmic reticulum of neurons in the central nervous system [10]. It was initially recognized as an antigen by anti‐myelin monoclonal antibodies [11], and later studies found that Nogo‐A is a neuronal growth inhibitor that can inhibit nerve regeneration after CNS injury [12]. Nogo‐A has two functional domains, Nogo‐66 loop and Nogo‐A‐Δ20, which are both located outside the cell [13]. Nogo‐A binds to NgR1 on the plasma membrane through the Nogo‐66 domain and then activates Rho‐associated protein kinase (ROCK) to induce an inhibitory effect on neurite outgrowth [14]. In previous work, we found that NgR activation not only inhibited neurite outgrowth but also promoted the production of Aβ [15]. Another study also showed that amyloid plaques and phosphorylated tau levels were ameliorated by inhibiting Nogo/NgR signaling in APP/PS1 mice [16] and that knocking out Nogo expression in APP transgenic mice can improve their learning ability and memory according to the Morris water maze (MWM) behavior [17]. These results suggest that Nogo/NgR1 plays an important role in the pathological process of Alzheimer's disease.

In a previous study, we identified a specific NgR1 antagonist peptide by using phage display technology [18]. NAP2 abrogates the inhibitory effects of Nogo‐66 on neurite outgrowth in PC12 cells and CGCs by preventing the activation of ROCK induced by Nogo‐66 in vitro [18]. Notably, owing to its small molecular weight, NAP2 exhibits a superior ability to cross the blood–brain barrier compared to other macromolecular proteins, thereby conferring significant advantages in the treatment of central nervous system diseases [19]. The primary objective of this study was to investigate whether NAP2 exerts therapeutic effects on Alzheimer's disease model mice and to explore its potential future applications in AD treatment. Specifically, we aimed to observe the impact of NAP2 on the learning and memory abilities of APP/PS1 model mice and to elucidate the underlying mechanisms. We anticipate that this study will make a meaningful contribution to the ongoing exploration of novel AD drugs that target amyloid deposition and promote neural regeneration.

## 2 Results

### 2.1 Liposome Encapsulation of NAP2 and Distribution of NAP2 in the Cortex and Hippocampus by Nasal Feeding and Intravenous Administration

**FIGURE 1:**
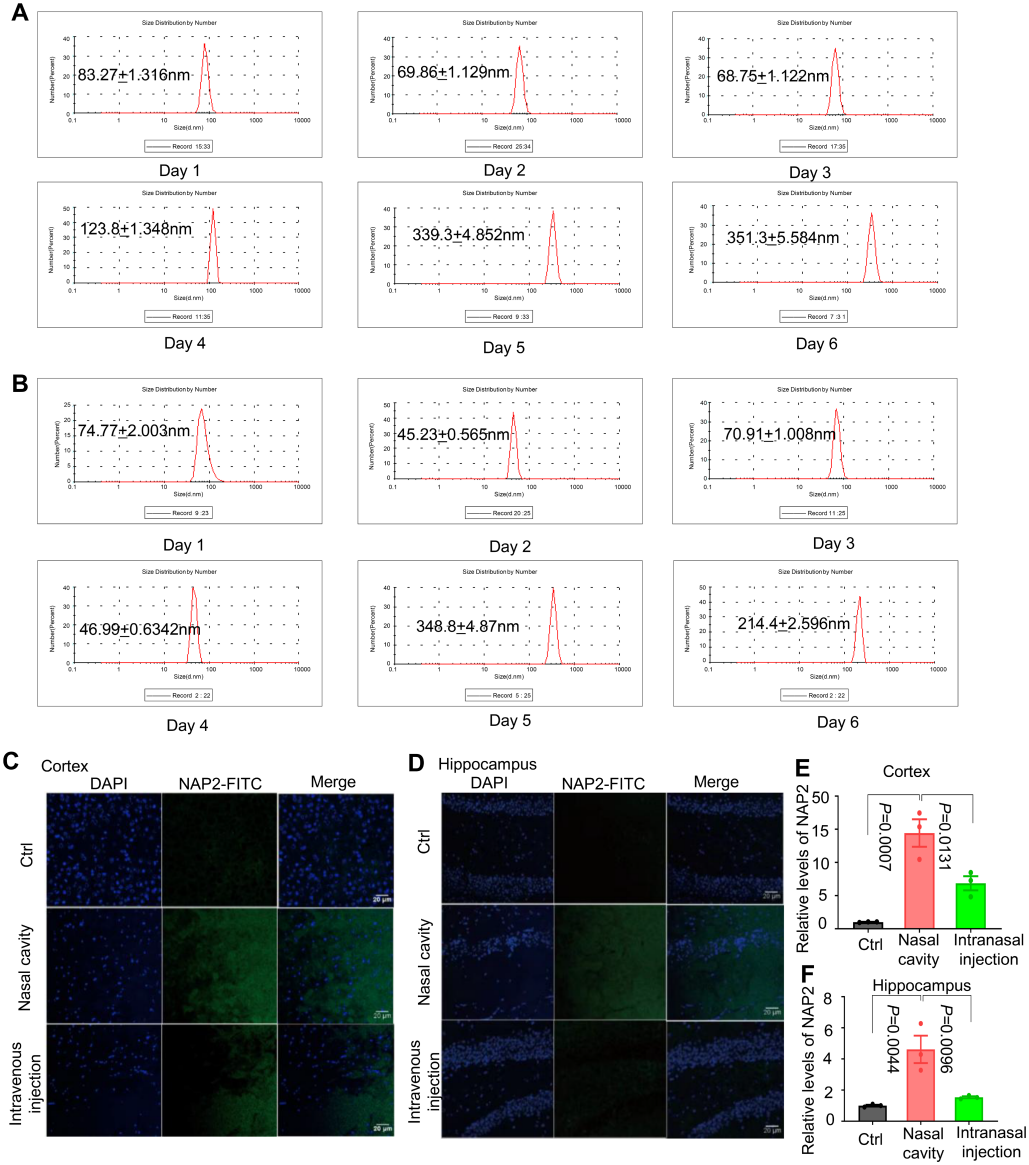
Characterization and brain distribution of NAP2 liposomes. (A, B) Distribution of empty liposomes (A) and NAP2‐encapsulated liposomes (B). (C–F) Representative images showing the distribution of NAP2 in the cortex (C) and hippocampus (D). Quantitative statistical analysis of NAP2 levels in the cortex (E) and hippocampus (F). The values are expressed as the mean ± SEM and analyzed by one‐way ANOVA and repeated measures ANOVA.

Liposomes are novel drug delivery systems with phospholipid bilayer structures that enable them to be highly compatible with the lipid layer of the blood–brain barrier, facilitating NAP2 delivery into the brain. The particle size distribution of the blank liposomes and drug‐loaded liposomes was measured for 6 consecutive days (Figure 1). The results showed that the particle size was < 100 nm in the first 3 days but > 100 nm on Day 4. This result suggested that the liposomes exhibit superior stability and dispersibility during the first 3 days (Figure 1A). During the first 3 days, the particle size of the drug‐loaded liposomes remained relatively stable and small, indicating suitable dispersion of the drug‐loaded liposomes (Figure 1B). The results demonstrate that, compared to the blank control group, mice administered FITC‐labeled NAP2 via tail vein injection exhibited green fluorescence in the cortical and hippocampal regions. Similarly, mice in the intranasal administration group showed pronounced green fluorescence in these brain regions, indicating that both administration routes enable NAP2 to penetrate the brain (Figure 1C,D). Importantly, quantitative analysis revealed that the relative levels of NAP2 in both the cortex and hippocampus were significantly higher following intranasal administration compared to intravenous injection (Figure 1E,F). These findings suggest that intranasal delivery is a more efficient route for targeting NAP2 to the brain. Therefore, subsequent animal experiments will adopt the intranasal administration route.

### 2.2 NAP2 Alleviates Learning and Memory Loss in APP/PS1 Transgenic Mice

**FIGURE 2:**
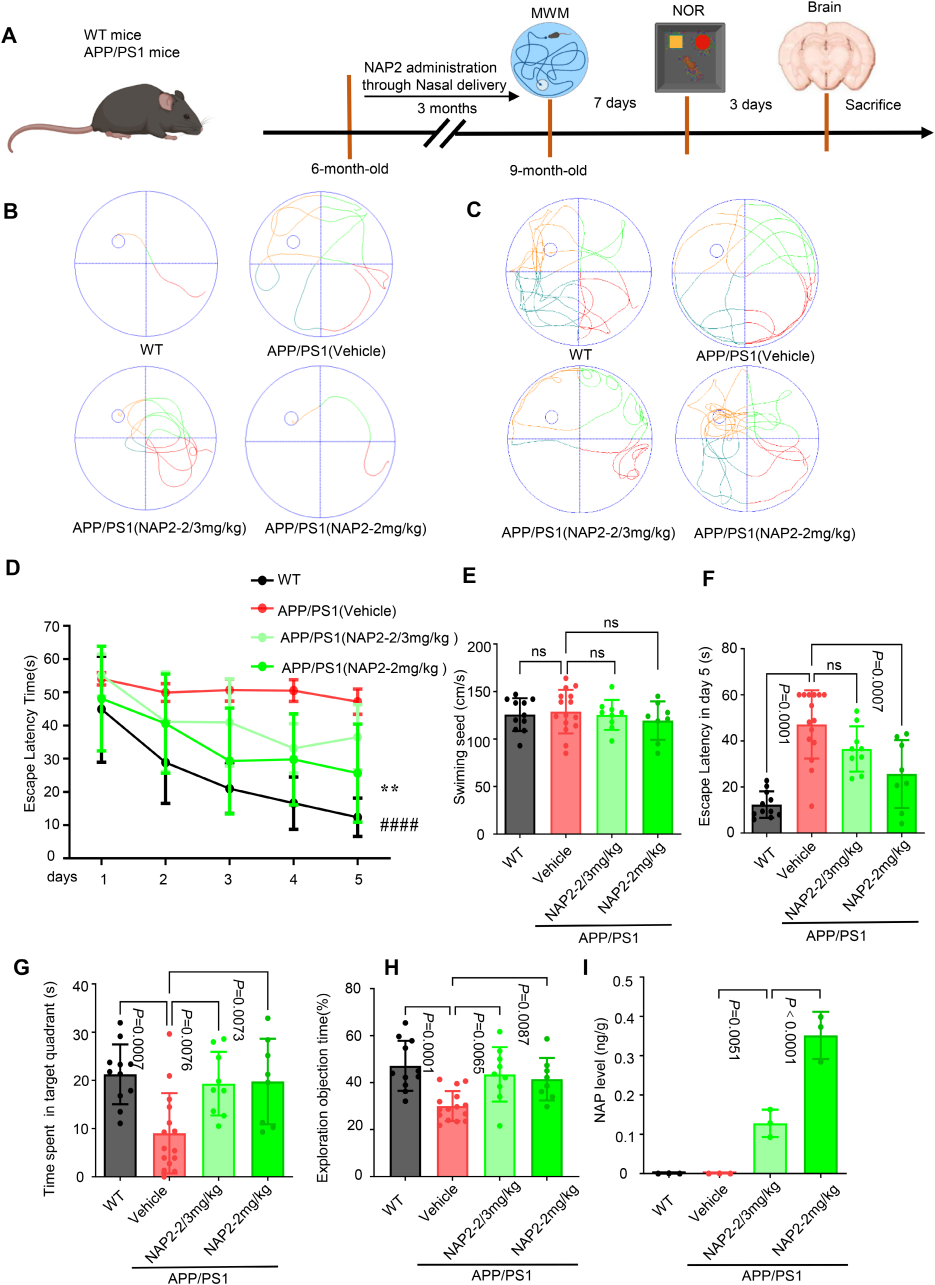
NAP2 improves the learning and memory abilities of APP/PS1 mice in the Morris water maze and the novel object recognition (NOR) tests. (A) Experimental schedule for APP/PS1 rats injected with NAP2 peptide. (B) Representative tracks for latency trail. (C) Representative tracks for space exploration trail. (D) The latency to reach the hidden platform during successive days of training in the Morris water maze was measured, and the results were quantified on the fifth day. (E) Mouse water maze swimming speed. (F) Escape latency of each group on Day 5. (G) Duration of time spent within the target quadrant for each group. (H) The statistical analysis of exploration time was conducted in the novel object recognition test. (I) Quantification of NAP levels (ng/g) in brain tissues among the different experimental groups. The values are expressed as the mean ± SEM and analyzed by one‐way ANOVA and repeat measured ANOVA. Repeat measured ANOBA was used in latency time from WMW behavior. *n* = number of rats, *n* = 11 for control, *n* = 15 for model, *n* = 9 for NAP2(2/3 mg/kg) group, *n* = 8 for NAP2 (2 mg/kg/group).

The amyloid plaques in the cerebral cortex of APPswe/PSEN1dE9 mice begin to appear by approximately 4 months of age [20]. Subsequently, cognitive deficits are detected between 6 and 10 months of age and worsen with age [21]. To explore whether NAP2 can alleviate cognitive impairment in APP/PS1 transgenic mice, the spatial learning and memory abilities of APP/PS1 mice were assessed using the Morris water maze (MWM) and novel object recognition (NOR) tests after 3 months of treatment with NAP2 (Figure 2A). According to the results of the navigation test in MWM, the escape latency of the model group was significantly longer than that of the control group on Day 5. Additionally, NAP2 (2 mg/kg) promoted the learning and memory abilities of APP/PS1 transgenic mice in the MWM test (Figure 2B,D–F). In the space exploration test, the results showed that memory dysfunction observed in APP/PS1 mice was alleviated in APP/PS1 (NAP2‐2/3 mg/kg) mice and APP/PS1 (NAP2‐2 mg/kg mice) (Figure 2C,F,G). Spatial memory was also assessed using a novel object recognition test. Mice were trained as mentioned above. As opposed to model mice, APP/PS1 (NAP2‐2/3 mg/kg) and NAP2 (2 mg/kg) mice were able to distinguish the old object within 24 h, whereas model mice failed to do so (Figure 2H). Furthermore, quantitative analysis confirmed the successful delivery and accumulation of the peptide, as evidenced by significantly elevated NAP levels in the treated APP/PS1 mice compared to the vehicle group. Notably, the NAP concentration in the NAP2‐2 mg/kg group was significantly higher than that in the NAP2‐2/3 mg/kg group (Figure 2I). Based on these results, NAP2 significantly attenuates cognitive behavioral deficits in APP/PS1 mice.

### 2.3 NAP2 Reduces Aβ Plaques Deposition in APP/PS1 Transgenic Mice

Alzheimer's disease is a devastating neurodegenerative disorder, and Aβ plaques are widely recognized as the primary initiator of the pathological cascade in AD, which ultimately leads to neuronal apoptosis through a series of intricate events [22]. Building on this understanding, we first evaluated the long‐term effects of NAP2 treatment on neural architecture. H&E staining of paraffin‐embedded brain sections revealed no obvious pathological changes, neuronal loss, or structural abnormalities across the groups, indicating that NAP2 treatment does not adversely affect overall brain morphology (Figure S1A–D).

**FIGURE 3:**
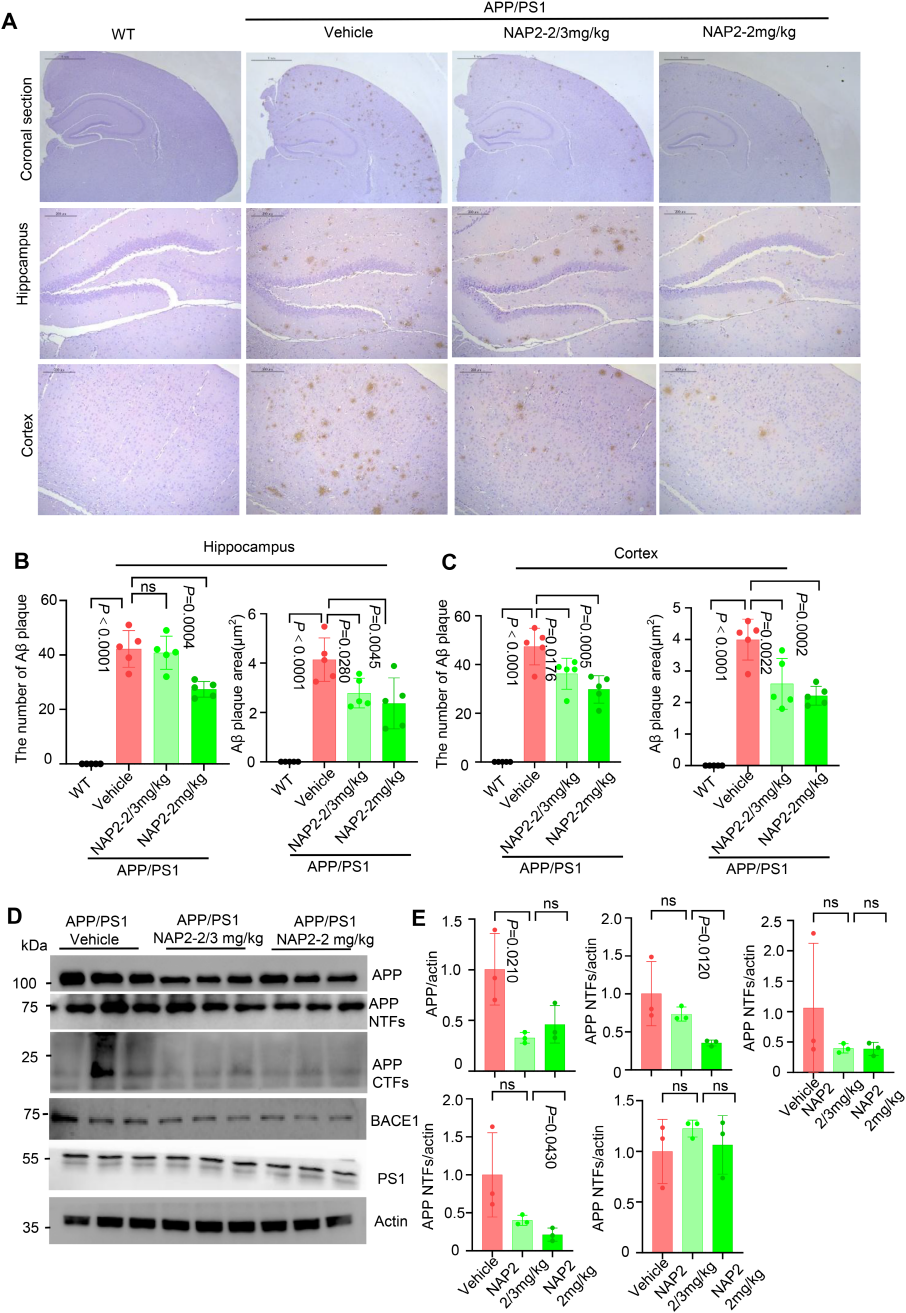
NAP2 Attenuated cerebral amyloid deposits and reduced the levels of Aβ in the brains of APP/PS1 mice. (A) Representative immunohistochemical staining images of the cerebral cortex and hippocampus (scale bar in coronal section: 1 mm; scale bar in HIP and cortex:200 μm). (B) The statistical analysis of Aβ plaque number and area in hippocampus (*n* = 5). (C) The statistical analysis of Aβ plaque number and area in cortex (*n* = 5). (D) Representative Western blot images showing the protein expression levels of APP, APP NTFs, APP CTFs, BACE1, and PS1 in the brain tissues of APP/PS1 mice across different treatment groups. Actin was used as an internal loading control. (E) Quantitative densitometric analysis of the relative protein levels of APP, APP NTFs, APP CTFs, BACE1, and PS1 normalized to Actin. The values are expressed as the mean ± SEM and analyzed by one‐way ANOVA.

Following this initial safety confirmation, we further investigated the impact of NAP2 on Aβ accumulation, a primary pathological hallmark of AD. Immunohistochemical staining of coronal brain sections revealed extensive Aβ plaque deposition in the hippocampus and cortex of vehicle‐treated APP/PS1 mice, whereas no plaques were observed in wild‐type mice (Figure 3A). Notably, NAP2 administration effectively alleviated this amyloid burden. Quantitative analysis demonstrated that both the number and the total area of Aβ plaques were significantly reduced in the APP/PS1 mice treated with NAP2 (2/3 and 2 mg/kg) compared to the vehicle group, in both the hippocampus (Figure 3B) and the cortex (Figure 3C). To further elucidate the mechanisms underlying this reduction in Aβ plaques, we investigated the effect of NAP2 on the Aβ production pathway. Western blot analysis (Figure 3D) and subsequent quantification (Figure 3E) revealed that NAP2 treatment significantly downregulated the protein levels of full‐length amyloid precursor protein in the brains of APP/PS1 mice. Concurrently, the levels of APP cleavage products, such as APP N‐terminal fragments (APP NTFs), were also markedly reduced following NAP2 administration. In addition, NAP2 treatment significantly reduced the expression of β‐site APP‐cleaving enzyme 1 (BACE1), a key β‐secretase responsible for amyloidogenic APP cleavage, whereas no significant change was observed in presenilin‐1 (PS1), a core component of the γ‐secretase complex (Figure 3D,E). Together, these findings suggest that NAP2 attenuates Aβ accumulation primarily by suppressing APP expression and its subsequent amyloidogenic processing.

### 2.4 NAP2 Mitigates Neurons and Dendritic Spines Loss in the Hippocampus of APP/PS1 Transgenic Mice

The role of NAP2 in enhancing memory function was further assessed by performing a transcriptome analysis of total mRNA obtained from APP/PS1 treated with NAP2 (2 mg/kg). Initially, the expression of the acquired genes was clustered into 16 groups, each representing various stages of gene expression changes from control to modeling and administration (Figure S2). Subsequently, Gene Ontology enrichment analysis was performed on the genes in these 16 clusters. The displayed data is only a subset of the complete dataset.

**FIGURE 4:**
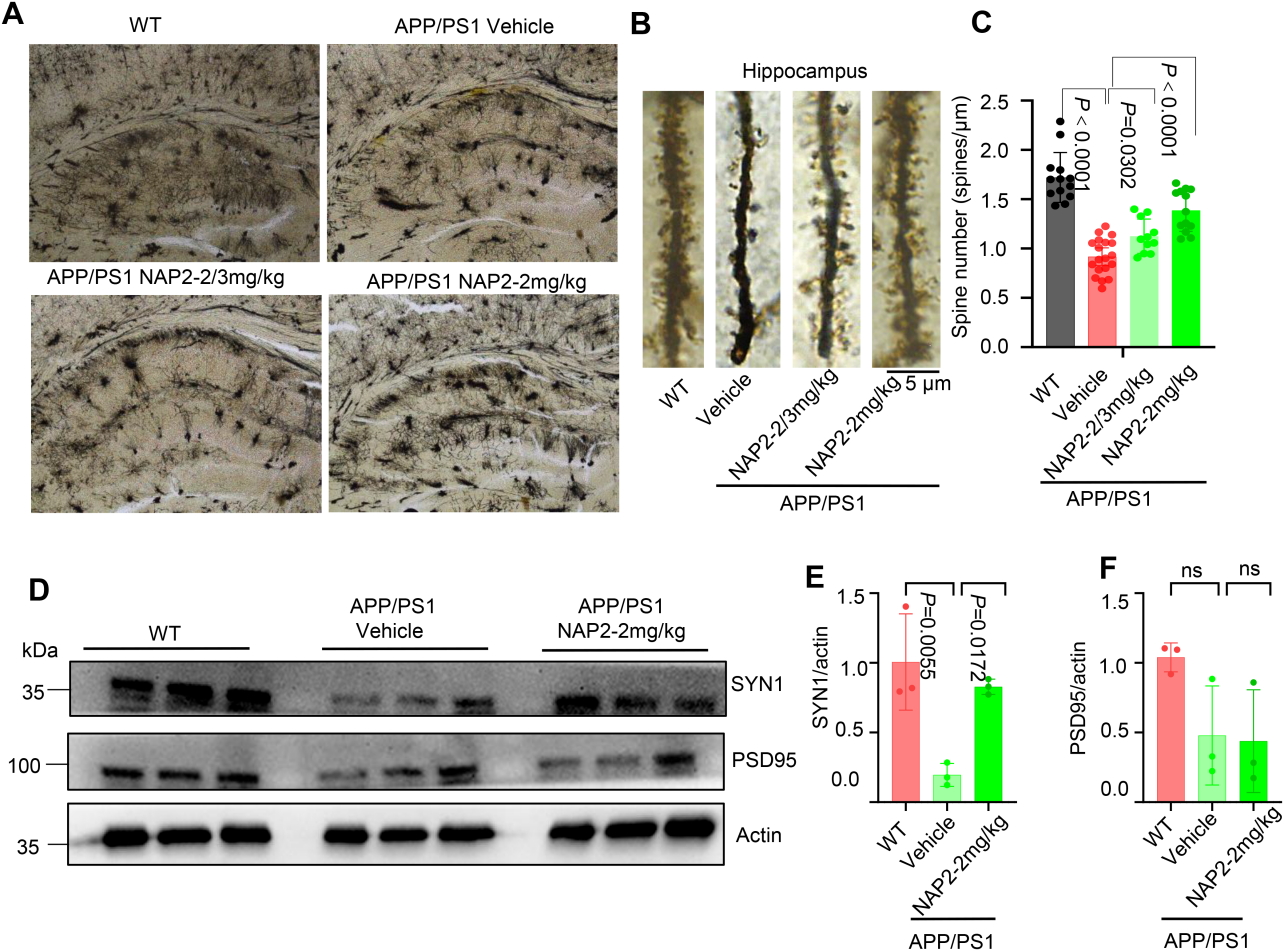
NAP2 hinders dendrite spines declining in the hippocampus of APP/PS1 mice. (A) The Golgi staining of hippocampus and DG in various experimental groups. (B) Representative images of dendrites from the dentate gyrus in various experimental groups. (C) The statistical analysis of spine number (*n* = 12). (D) Western blot analysis was utilized to detect the levels of synapsin1 and PSD95 in NAP2 treatment. (E, F) Graph representing the relative phosphorylation level synapsin1 and PSD95 detected from Western blot analysis. The quantitative statistics data are from triplicate samples, presented as the mean ± SEM.

Our results suggested that many of these clusters, through the control‐model‐administration changes, primarily exhibited GO enrichment associated with RNA splicing and mRNA changes, without significant implications. However, an interesting finding was observed in Cluster 9, 12 and 16, where the expression of certain genes was low in the control group, high in the model group, and low again in the NAP2‐treated (2 mg/kg) administration group. The GO enrichment indicated that these genes were related to mitochondrial membrane organization, axonogenesis, synapse organization, and gliogenesis. Furthermore, Golgi staining was used to analyze the effect of NAP2 on the neurites of hippocampal neurons. The number of hippocampal neurons and dendrite spines decreased in APP/PS1 mice compared with the WT mice (Figure 4A). Compared to APP/PS1 mice, the NAP2‐treated (2 mg/kg) in APP/PS had an increased number of hippocampal neurons and a higher density of axons and dendrites. The density of dendritic spines in the hippocampus of APP/PS1 mice was significantly lower than that in the WT mice. In contrast, the density of dendritic spines in the NAP2‐treated (2 mg/kg) group was significantly improved compared with that in the APP/PS1 mice (Figure 4B,C). NAP2 also significantly upregulated the expression of synapsin1 and PSD95 protein, which was in line with the results of Golgi staining (Figure 4D,F). These data revealed that NAP2 administration can mitigate amyloid toxicity in APP/PS1 mice while also enhancing the growth of neuronal dendrites and increasing the number of spines in the hippocampus of APP/PS1 mice.

### 2.5 NAP2 Exerts Inhibitory Effects on Aβ_42_‐Triggered Mitochondrial Dysfunction In Vitro

**FIGURE 5:**
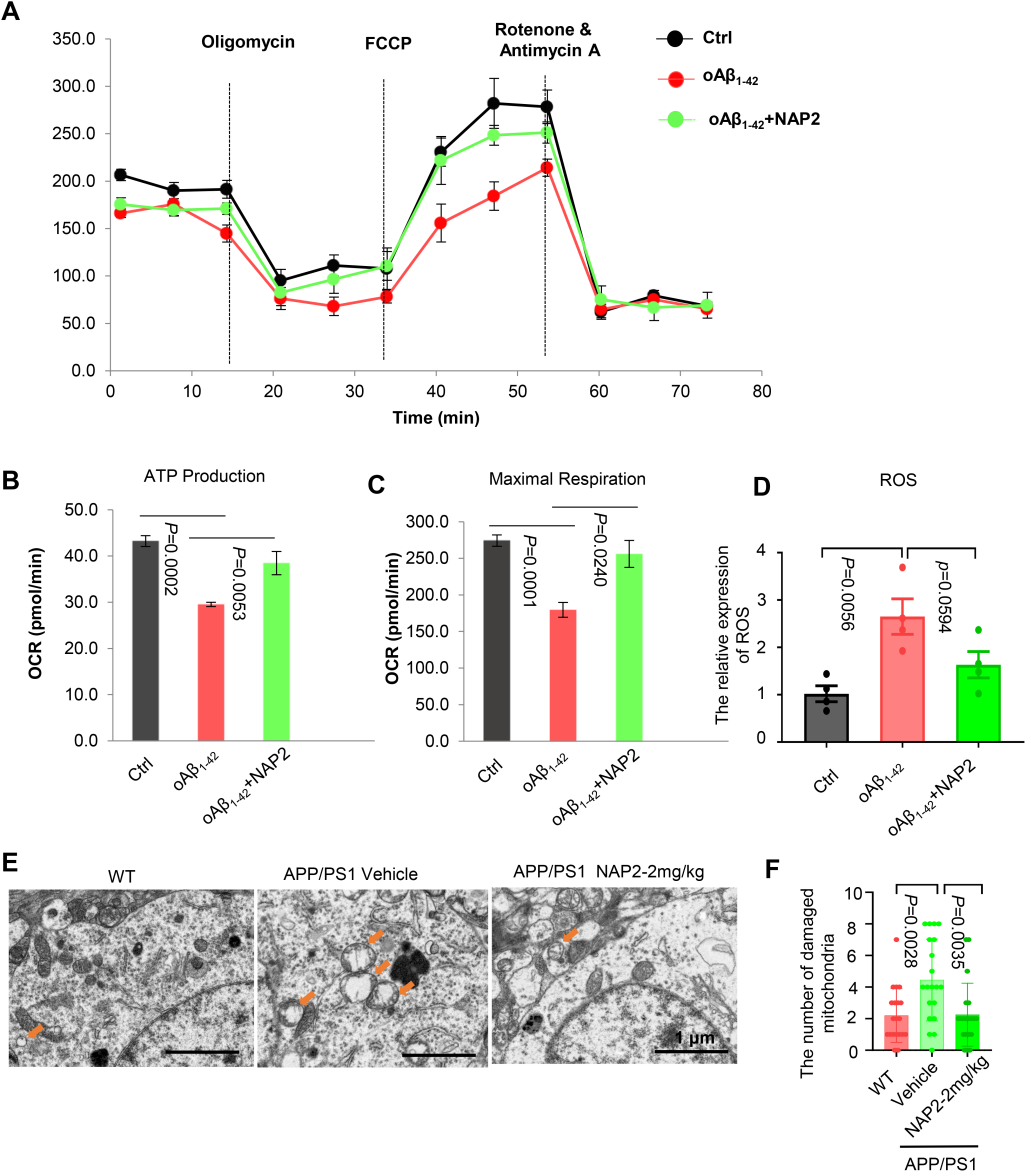
NAP2 ameliorates mitochondrial dysfunction induced by Aβ42 and decreased ROS levels. (A) Seahorse assay measuring OCR in HT22 cells treated with or without Aβ42 and NAP2. (B) Relative changes in oxygen consumption after FCCP treatment of HT22 cells. (C) Quantification of the difference between maximal respiratory capacity after addition of substrates specific respiratory capacity after complete inhibition of the respective complex. (D) ROS generation was measured using a ROS assay kit. (E) Representative transmission electron microscopy images showing mitochondrial morphology in the brain tissues of WT, vehicle‐treated APP/PS1, and NAP2‐treated (2 mg/kg) APP/PS1 mice. Orange arrows indicate damaged mitochondria (characterized by swelling and disrupted or lost cristae). Scale bar = 1 μm. (F) Quantitative analysis of the number of damaged mitochondria across the different experimental groups. The values are expressed as the mean ± SEM and analyzed by one‐way ANOVA.

The compromised bioenergetic capacity of mitochondria is widely recognized as a fundamental pathological mechanism underlying Alzheimer's disease neurodegeneration. Here, Figure 4F illustrates the oxygen consumption rates (OCR) of HT‐22 cells subjected to treatment with oligomeric Aβ42 with or without NAP2 co‐treatment. The findings revealed a significant decrease in ATP production and maximum capacity OCR in HT‐22 cells treated with oligomeric Aβ42 alone. Conversely, cells co‐treated with Aβ42 and NAP2 exhibited higher levels of ATP production and maximum capacity OCR compared to those treated only with Aβ42 (Figure 5A–C). Additionally, the administration of NAP2 showed a tendency to mitigate the production of reactive oxygen species (ROS), although these findings were not statistically significant (Figure 5D).

To further validate the protective effects of NAP2 on mitochondria in vivo, we examined the mitochondrial ultrastructure in the brains of APP/PS1 mice using transmission electron microscopy. As shown in Figure 5E, mitochondria in the vehicle‐treated APP/PS1 mice exhibited severe morphological damage, characterized by swelling and disrupted or absent cristae (indicated by orange arrows), compared to the intact mitochondria observed in WT mice. Notably, NAP2 treatment (2 mg/kg) markedly preserved the mitochondrial architecture. Quantitative analysis (Figure 5F) confirmed that the amount of mitochondria damage was significantly elevated in the APP/PS1 vehicle group, and this structural impairment was substantially reversed by NAP2 administration. Therefore, collectively, these functional and structural findings suggest that NAP2 attenuates mitochondrial dysfunction caused by Aβ42‐induced oxidative stress and structural damage.

### 2.6 NAP2 Reduced Aβ Levels and Tauopathy by Suppressing the NgR/ROCK Signaling Pathway

**FIGURE 6:**
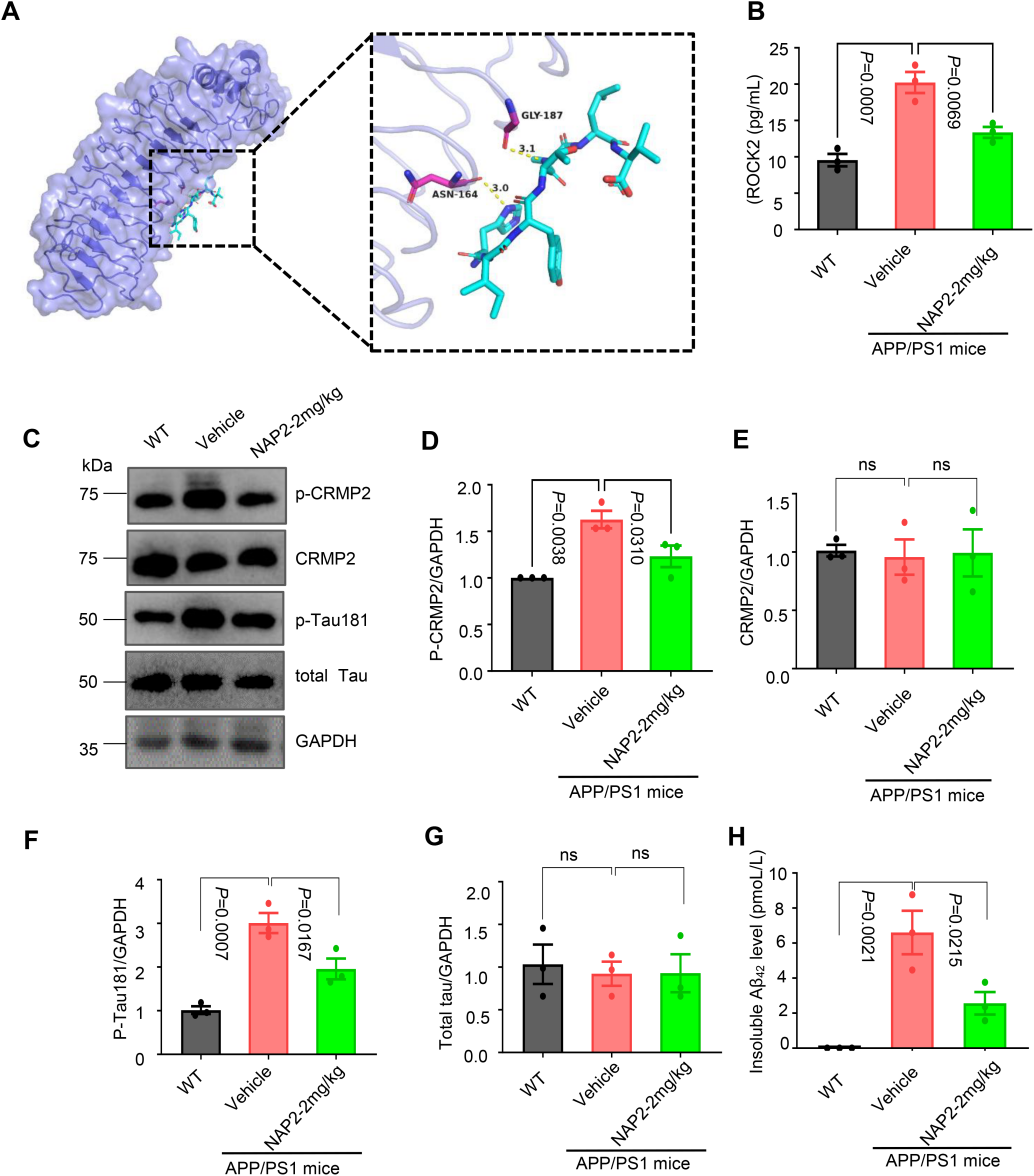
Molecular docking analysis of NAP2 to NgR and NAP2 reduce the Aβ42 and p‐tau via NgR/ROCK2 pathway. (A) The interactions between NgR and NAP2. (B) Level of ROCK2 in the hippocampus of different groups were detected using ELISA assay. (C–G) Western blotting was used to analyze the CRMP2 and tau protein expression in NAP2‐2 mg/kg treated APP/PS1 mice. (H) The hippocampal insoluble Aβ42 expression was quantified using the ELISA method.

To explore the potential competitive binding of NAP2 to the Nogo receptor, we first conducted an in‐depth analysis of the interactions between NgR and NAP2. By employing relevant techniques, we successfully identified and classified all functional residues based on their interaction patterns. It was found that multiple groups of residues were involved in the interactions between the receptor protein and the ligand. For instance, a hydrogen bond formed between GLY187 of NgR and the ligand (Figure 6A). Based on these interaction forces, the binding energy of the protein‐ligand complex was calculated to be –6.0 kcal/mol, indicating a strong binding affinity between NgR and NAP2. RhoA and Rock are well known downstream molecules that play a crucial role in regulating the inhibitory effect of Nogo‐A/NgR [23]. Building on this knowledge, we further investigated whether the ROCK2‐CRMP2 signaling pathway contributes to the effect of NAP2 on Aβ deposition and neuron regeneration. Through ELISA assays, we observed that ROCK2 expression in the model group was significantly higher than that in the control group. In contrast, treatment with NAP2 (2 mg/kg) led to a down‐regulation of ROCK2 expression (Figure 6B). Moreover, the reduction in CRMP2 phosphorylation by NAP2 was in line with the observed trend of Aβ expression (Figure 6D,E,H).

Due to the hyperphosphorylation of tau, the accumulation of NFTs is recognized as an early indicator of AD [24]. To assess the impact of NAP2 on tau phosphorylation in APP/PS1 mice, we performed Western blot analysis to quantify the levels of tau expression and phosphorylation in the hippocampus. Compared with the model group, both the control group and NAP2 (2 mg/kg) group exhibited a significant reduction in tau phosphorylation (Figure 6F,G). These findings strongly suggest that the NgR/ROCK2 pathway may play a pivotal role in Aβ deposition and tau hyperphosphorylation. NAP2 appears to promote the learning and memory ability of APP/PS1 mice by blocking NgR/ROCK2 signaling.

## 3 Discussion

Substantial progress has been made in elucidating the molecular and cellular processes associated with AD. Aβ plaques and NFTs (neurofibrillary tangles) lead to a cascade of reactions that ultimately cause synaptic dysfunction and neuronal death, which results in memory decline and cognitive impairment [25]. To date, therapeutic strategies are still mainly focused on Aβ and include promoting Aβ degradation and decreasing Aβ production. Nogo‐A is considered the most potent inhibitor of neuronal regeneration among myelin‐derived molecules, including MAG and OMgp [26]. Nogo‐A and Nogo‐66 receptor 1 have been found to be overexpressed in the pyramidal neurons of the hippocampus in Alzheimer's patients, especially in the CA1 and CA2 regions and near senile plaques [22, 27]. Several studies have shown that Nogo/NgR is associated with Aβ production. A reduction in NgR leads to a decrease in Aβ peptide production and improves synaptic function in APP/PS1 mice [28], and NgR impairs the clearance of fibril Aβ by microglia [29]. These studies suggested that finding a specific antagonist of NgR could both abrogate the inhibition of neural regeneration and reduce the production of Aβ. Blocking the interactions between Nogo‐A and its receptors may be a novel strategy for the treatment of Alzheimer's disease [30].

A previous study reported that liposomes can facilitate the passage of drugs across the BBB in a Tg2576 mouse model [31]. In our study, NAP2 was loaded into liposomes, and the intranasal administration route was used to aid its transport across the BBB. Changes in synaptic structure and function and loss of synapses and neurons can lead to neurological dysfunction, which aggravates cognitive dysfunction [32]. Synaptic damage and loss of nerve endings occur prior to neuronal cell death and are a main biological factor leading to memory and cognitive dysfunction during AD and other types of dementia [33]. Aβ is particularly toxic to neurons, and the area surrounding Aβ plaques shows the most damage to synapses and neurites [34]. In our study, Aβ42 and amyloid plaque deposition in the hippocampus, dentate gyrus, and cortex was significantly reduced, while the density of axons and dendrites increased in the hippocampus, which corresponded with cognitive improvement in behavioral tests. Importantly, our molecular analysis revealed that this reduction in Aβ burden is driven by the downregulation of full‐length APP and its cleavage products (APP NTFs). It is suggested that NAP2 may exert a neuroprotective effect on synapses and dendrites through its potential to attenuate amyloid genesis at the source.

Aβ42 peptides aggregate to form Aβ oligomers, which induce ROS production and Ca2+ influx into neurons through postsynaptic N‐methyl‐D‐aspartate receptors (NMDARs) and then cause mitochondrial dysfunction. Furthermore, the presence of dysfunctional mitochondria enhances the intracellular accumulation of ROS, ultimately leading to neuronal cell death [35, 36]. Studies have also demonstrated that oxidative stress plays a pivotal role in promoting tau phosphorylation and the formation of neurofibropathies under pathological conditions. In addition, p‐tau can also enhance the generation of ROS [37, 38]. Repetitive synaptic damage resulting from insufficient mitochondrial ATP levels contributes to the progressive degeneration of synapses [39]. Our study showed that NAP2 has the potential to mitigate mitochondrial dysfunction and oxidative stress induced by Aβ oligomers in vitro. Furthermore, our in vivo ultrastructural analysis using TEM directly confirmed that NAP2 administration significantly preserves mitochondrial morphology, rescuing them from severe swelling and cristae disruption in APP/PS1 mice. These findings were consistent with the snRNA‐seq data showing that NAP2 administration attenuated changes in the nervous system, including synapse organization.

Nogo‐A binds to its receptors NgR1 and p75NTR and acts as a link in transmembrane signaling by activating the RhoA/ROCK pathway. ROCK regulates the activity of its substrates MLC, LIMK‐1, and CRMP2 followed by the inhibition of neurite outgrowth, synaptic plasticity, growth cone collapse, and the generation and phosphorylation of tau [40]. The phosphorylation of CRMP‐2 may play a key role in axonal degeneration downstream of NgR1 activation [41]. In this study, the results showed that NAP2 liposomes could significantly reduce ROCK2 expression levels and CRMP2 phosphorylation in vivo, which was consistent with our previous study in vitro [16]. There is mounting evidence that Aβ42 can activate the RhoA/ROCK signaling pathway, which causes synaptic loss [42, 43]. Furthermore, ROCK2 activation can increase β‐secretase cleavage of APP, which causes Aβ accumulation, resulting in a detrimental feedback loop [44, 45]. A study showed that statins can reduce the expression level of Aβ in cells by decreasing the activity of ROCK and inhibiting the RhoA/ROCK pathway [46]. Jeremy H et al. found that ROCK2 inhibition in AD model mice downregulated the expression of Aβ [45]. A study reported that Fasudil reduces Aβ production through inhibition of the NgR/ROCK2 pathway in APP/PS1 mice. The findings of our study indicate that the inhibition of NgR/ROCK signaling through the use of NAP2 leads to a reduction in Aβ production. These results provide evidence supporting the notion that the termination of the positive feedback loop between Aβ and ROCK can be achieved by inhibiting ROCK2.

In addition to Aβ, NFTs are another major neuropathological hallmark of AD. They are formed by tau hyperphosphorylation and abnormal aggregates [47]. Specifically, phosphorylation of Tau at threonine 181 (p‐Tau181) is recognized as a crucial driver of AD pathology, which directly correlates with amyloid burden and exacerbates synaptic toxicity, microtubule destabilization, and subsequent neuronal loss [48]. Previous research has demonstrated that the activation of ROCK can lead to an increase in the levels of phosphorylated tau protein. ROCK inhibitors, such as FSD‐C10 and thiazovivn, can reduce tau aggregation and p‐tau formation in vivo and in vitro, respectively. The inhibition of ROCK could also prevent tau phosphorylation after cerebral ischemia [49]. Furthermore, Aβ induces tau hyperphosphorylation by activating CKD‐5 and GSK‐3β [50]. Therefore, it may be inferred from this study that NAP2 reduces tau phosphorylation (particularly the pathological p‐Tau181 species) by directly inhibiting the NgR/ROCK pathway and indirectly reducing Aβ‐induced tau hyperphosphorylation, in turn improving the learning and memory ability of APP/PS1 transgenic mice.

Despite promising results with NAP2 in APP/PS1 mice, several limitations exist. The study employed a prevention paradigm rather than a treatment approach for established pathology, potentially limiting clinical relevance. While NAP2 crosses the blood–brain barrier, further pharmacokinetic characterization of liposome‐encapsulated NAP2 is needed. The molecular interactions between NAP2 and NgR1 were primarily assessed through computational modeling, requiring additional biophysical validation. Our investigation focused mainly on the NgR/ROCK2/CRMP2 pathway without extensively exploring potential off‐target effects. Another limitation is the use of HT22 cells, which, as immortalized mouse hippocampal neuronal cells, cannot fully mimic terminally differentiated neurons in the brain. Thus, the in vitro findings should be further validated in primary neurons or human iPSC‐derived neurons.

In conclusion, our study demonstrated that intranasal administration of the small‐molecule NgR antagonist peptide NAP2 effectively mitigated cognitive behavioral deficits, targeted Aβ production, rescued mitochondrial function, and promoted neurite regeneration. While NAP2 was shown to inhibit the NgR/ROCK pathway, further studies are required to fully elucidate the precise causal relationship between this signaling cascade and the clearance of Aβ. Nevertheless, these findings revealed the therapeutic potential of NAP2 in AD.

## 4 Materials and Methods

### 4.1 Liposome Preparation and Encapsulation of NAP2

The thin‐film ultrasound method was used to prepare liposomes [51] in this experiment. Hydrogenated soy lecithin and cholesterol (120 mg: 40 mg; Aiweituo, NO1003) were weighed and dispersed in 3 mL of chloroform. The mixture was subjected to rotation and evaporation at 30°C for 25 min to eliminate the organic solvents. Then, the mixture was placed in a fume hood to dry. PBS buffer (8 mL, 0.01 M) was used to dissolve the thin film. After sonicating for 1 h at 40°C, the solution was transferred to a 15 mL centrifuge tube and incubated in a water bath until all visible particles dissolved. Then, the liposome solution was stored at 4°C until needed.

NAP2 (Sequence: HIYTALV) was synthesized by the China Peptides Co. Ltd. (Shang‐hai, China). The purity of the peptides (98.31%) was analyzed by HPLC and mass spectrometry. After NAP2 (10 mg) and FITC‐NAP2 (10 mg) were dissolved in 1 mL liposomes, the liposome solution of control liposomes and dissolved drugs was diluted with 0.01 M PBS buffer to a certain concentration. A laser nanoparticle size analyzer was used to measure the particle size.

### 4.2 Detection of the Amount of Peptide in the Brain

NAP2 liposomes were labeled with fluorescein isothiocyanate (FITC) (GL Biochem, Shanghai) and dissolved in PBS and prepared into a solution under light‐protected conditions. Mice were divided into three groups, each containing three mice: a blank control group (administered PBS), an intranasal administration group, and an intravenous administration group, both of which received the pre‐set dose of NAP2‐FITC.

Mice were secured by gently pinching the skin behind the neck and shoulders and positioned in a supine position with the abdomen facing up. The neck and chin were aligned to form a straight line parallel to the floor, ideally close to a 180°angle. Using a 10 μL pipette, 3 μL of the prepared NAP2‐FITC liposomes solution or PBS was drawn, forming a droplet near one nostril. The droplet was allowed to be inhaled by the mouse's natural breathing without touching the nostril to prevent irritation and swelling. The mouse was maintained in this position for 15 s to ensure complete absorption. The process was repeated for each mouse in the group. After the initial administration, the same procedure was repeated for the other nostril. For the intravenous administration group, NAP2‐FITC (0.5 μg/μL) liposomes were administered via the tail vein in a total volume of 120 μL. The blank control group received an equivalent volume of PBS.

1 h after administration, brain tissues were harvested from the mice under light‐protected conditions. The tissues were embedded in OCT compound and frozen sections were prepared, maintaining light protection throughout the process. The sections were stained with DAPI for 10 min. Slides were mounted with an anti‐fade reagent and immediately imaged using laser confocal microscopy.

### 4.3 Animal Model Grouping and Drug Treatment

Adult male APP/PS1 double transgenic mice [B6C3‐TG (APPSWE, PSEN1DE9) 85DBO/J] and wild‐type mice were purchased from Nanjing Biomedicine Research Institute. All mice were housed in the specific pathogen‐free (SPF) room of the Experimental Animal Center of Jinan University under a 12‐h light–dark cycle, which complied with the EU Directive 201063EU guidelines for animal testing. APP/PS1 transgenic mice were randomly divided into low‐dose (2/3 mg/kg), high‐dose (2 mg/kg), and model groups by simple randomization.

At the age of 5 months, APP/PS1 mice were treated with different concentrations of NAP2 liposomes every day, except for the control and model mice. Mice in the control group and the model group were treated with liposomes without NAP2 by intranasal administration. The learning and memory ability of each group was tested by the Morris water maze and new object recognition test after 3 months of continuous administration. The operation of laboratory animals was in accordance with the regulations of the Animal Research Committee of the School of Medicine in Jinan University (approval No: IACUC‐20180120‐05).

### 4.4 Morris Water Maze Test

The MWM test was used to assess the learning and memory ability of mice after 3 months of NAP2 administration. The MWM experiment consisted of place navigation and spatial probe tests and was performed as previously described with necessary modifications [52]. The MWM device consists of a 100 cm high circular tank and a circular platform with a high‐definition camera that is connected to a computer to record the mice. The pool of water was divided into four quadrants: I, II, III, and IV. The platform was located in the second quadrant. Each group of mice entered the water from different quadrants (I, II, III, and IV). The track of the mice seeking and climbing to the platform from the entrance and the time of escape latency were automatically recorded by the behavioral analysis system (Shanghai Jiliang Technology, DigBehv‐MM). If the mouse could not find a platform within 60 s, the escape latency was recorded as 60 s, the researcher guided the mice to the platform, and the mouse was left on the platform for 15 s. The entry quadrant was changed randomly each day, and the mice were trained for 5 days (Day 1–5). Following the place navigation test, the platform was removed, and the mice were put in the water from the wall of quadrant IV. The spatial learning ability of the mice was determined by the latency time taken to find the platform. The software recorded the movement track of the mice for 60 s and analyzed the time spent in target quadrant II where the original platform was placed.

### 4.5 Novel Object Recognition Test

The NOR test was used to test mouse recognition memory as stated previously [53]. The testing process was divided into three phases: habituation, sample, and testing. The NOR test was conducted in a white box (40 cm × 40 cm × 40 cm) with a high‐definition camera connected to the computer to track the movement of the mice. The temperature of the room was set at 24°C ± 1°C throughout the test. During the habituation phase, the mice were placed in the box for 10 min to adapt to the surroundings before being sent back to their enclosure for 1 h of rest. In the sample phase, two yellow ping‐pong balls were placed on the same side of the box, 10 cm away from the wall of the box. The mouse was placed in the arena containing the two balls and given 10 min to explore before being returned to their home cage. 24 h later, one of the ping‐pong balls was replaced by a purple block of similar size. The mice were placed between the yellow ping‐pong ball and the purple block and were allowed to explore the objects freely for 10 min. Memory performance was determined by the time spent exploring novel objects. The data were collected and analyzed using the video tracking program Topscan 3.0 software (Clever Sys Inc.).

### 4.6 Hematoxylin–Eosin (H&E) Staining

A Hematoxylin and Eosin Staining Kit (Beyotime) was used to stain the tissue with hematoxylin–eosin (H&E). Briefly, the brain hemispheres of mice were fixed with 4% paraformaldehyde. Then, the samples were embedded in paraffin and cut into sections (thickness of 4 μm), followed by dewaxing and hydration. After staining with hematoxylin for 10 min, the sections were immersed in a 70% ethanol solution of 1% HCl for a few seconds and then washed with 0.01 M PBS solution. Subsequently, sections were stained with eosin solution for 2 min. Finally, the sections were dehydrated and sealed with neutral resin. An optical microscope (IX71, Olympus) was used to capture the images in the hippocampal and cortical regions.

### 4.7 Preparation of Aβ Oligomers

Aβ1‐42 peptide was dissolved in HFIP, evaporated under nitrogen gas, and dried to form a peptide film. The film was resuspended in DMSO and diluted to 100 μM in cold PBS/F12 medium. After incubating at 4°C for 24 h to allow oligomerization, the solution was centrifuged at 14,000 × *g* for 10 min at 4°C. The supernatant containing Aβ oligomers was collected for immediate use.

### 4.8 Cell Culture

HT‐22 cells (a mouse hippocampal neuron cell line) were cultured in DMEM supplemented with 10% fetal bovine serum at 37°C in 5% CO_2_. The cells were exposed to 5 μM Aβ oligomers and 2 mg/kg NAP2 for approximately 48 h prior to subsequent analysis.

### 4.9 Mitochondrial Respiration

Oxygen consumption rates (OCR) were measured using an XF‐24 Extracellular Flux Analyzer (Seahorse Bioscience) following the instructions. Briefly, HT‐22 cells were seeded onto Seahorse XF24 culture plates at a density of 5000 cells/well and incubated for 24 h. All media and reagents were adjusted to pH 7.4 before the experiment. After four baseline measurements, cells were sequentially challenged with the following inhibitors: oligomycin, fluoro‐carbonyl cyanide phenylhydrazone (FCCP), rotenone, and antimycin A. OCR values are shown as the mean ± SEM.

### 4.10 Immunohistochemistry

First, the paraffin‐embedded sections were prepared as described above. Sections were deparaffinized, rehydrated, and blocked. Then, the sections were incubated with primary Aβ antibody (Cell Signaling Technology, 9888, 1:1000, rabbit mAb) overnight at 4°C. The next day, the sections were incubated with the secondary antibody HRP‐conjugated goat anti‐rabbit IgG (Boster Biotech, BA1056, 1:1000, goat mAb) at room temperature for 30 min in the dark, and then 300 μL DAB reagent (Boster Biotech, AR1022) was added for staining. Finally, the sections were dehydrated after being stained with hematoxylin (Shanghai Yuanye, B25380). The quantity and area of Aβ plaques were counted in the picture by using Image‐Pro Plus software.

### 4.11 ELISA for Insoluble Aβ1‐42, ROCK and ROS

Mouse whole brain tissues were homogenized in 8 volumes of ice‐cold TBS containing 5 mM EDTA, 2 mM 1,10‐phenanthroline, and protease/phosphatase inhibitors [106]. Homogenates were centrifuged at 100,000 × *g* for 1 h at 4°C. To extract the insoluble Aβ fraction, the resulting pellets were homogenized in 70% formic acid (FA) and centrifuged again at 100,000 × *g* for 1 h at 4°C. The FA‐extracted supernatants were collected, neutralized with 1 M Tris‐base (pH 11; 1:20 *v*/*v*), and analyzed for Aβ1‐42 levels using a specific ELISA kit (Wako, #294‐62501) according to the manufacturer's instructions.

The brain hemispheres of mice were treated with RIPA lysis buffer containing protease inhibitor (200 μL lysate was added to every 20 mg tissue). The tissue was quickly cut into pieces with sterile scissors, and then ultrasound was applied to the cell crusher 3 times, each time at a 30 s interval of 1 min. Then, the suspension was centrifuged at 12000 rpm for 15 min. The supernatant was transferred to an EP tube for ELISA. According to the manufacturer's instructions, the levels of ROCK and reactive oxygen species were measured using an ELISA kit (Meimian, MM‐44832 M1; ROS Assay Kit‐Highly Sensitive DCFH‐DA R252). The plates were read at a wavelength of 450 nm using a UV spectrophotometer (PerkinElmer, Multiskan Sky Microplate). The total protein levels were calculated based on a standard curve.

### 4.12 Golgi Staining

The dendritic spines of neurons were detected by an FD Rapid GolgiStain kit (FD Neuro Technologies, USA). Briefly, the mouse brain was submerged in staining solution for 14 days. The brains were cut into 100 μm thick sections with a cryostat. Then, the glass extractor and solution C provided by the kit were used to transfer the sections to gelatin‐coated slides according to the manufacturers' protocol. After staining and dehydration, the sections were sealed with neutral resin. For spine density analysis, images of the hippocampus and DG were taken by an IX71 microscope (Olympus).

### 4.13 Molecular Docking

The X‐ray crystal structures of RTN4R (PDB: 5O0N) were retrieved from the Protein Data Bank. The protonation state of all the compounds was set at pH = 7.4, and the compound 3D structures were obtained using Open Babel [54]. AutoDock Tools (ADT3) were applied to prepare and parametrize the receptor protein and ligands. The grid maps were generated by AutoGrid, and AutoDock Vina (1.2.0) was used for docking simulation [55, 56]. The optimal binding pose was selected to analyze the interaction. Finally, the protein–ligand interaction figure was generated by PyMOL. The RTN4R protein is represented as a slate‐colored 3D model, the ligand is shown as a cyan‐colored ball‐and‐stick model, and their binding sites are shown as lines. The nonpolar hydrogen atoms were omitted. The hydrogen bonds, ionic interactions, and hydrophobic interactions are depicted as yellow, magenta, and green dashed lines, respectively.

### 4.14 Western Blot

The brain tissue was first homogenized in RIPA lysis buffer (Beyotime, P0013B) with a protease inhibitor cocktail (Sigma–Aldrich, P8340). Subsequently, the supernatant of the tissue homogenate was collected after centrifugation at 14000 × *g* at 4°C for 10 min. The protein concentration in the supernatant was determined using a BCA assay kit (BB‐3401, BestBio Inc.). The supernatant was mixed with 5× loading buffer to make a 2 μg/μL protein sample dilution. Equal amounts of proteins were separated by 10% sodium dodecyl sulfate–polyacrylamide gel electrophoresis (SDS–PAGE) and transferred to polyvinylidene fluoride (PVDF) membranes. The PVDF membranes were blocked with TBST with 5% (*w*/*v*) nonfat dried milk for 1 h and incubated with specific primary antibodies overnight at 4°C, including anti‐CRMP2 (CST 35672, 1:1000, rabbit mAb), anti‐GAPDH (ProteinTech, 10494‐1‐AP, 1:1000, rabbit mAb), anti‐p‐CRMP2 (CST 9397, 1:1000, rabbit mAb), anti‐tau (Abcam, ab76128, rabbit mAb), anti‐p‐tau (Abcam, ab109390, rabbit mAb), anti‐synapsin1 (CST, 5297, 1:1000, rabbit mAb), anti‐psd95 (Invitrogen, OSD00037 W, 1:1000, rabbit mAb), and anti‐β actin (CST, 3700, 1:2000, rabbit mAb). The membranes were then incubated with the appropriate HRP‐conjugated goat anti‐rabbit IgG secondary antibody (Earthox, E030120‐01, 1:7000, goat mAb) for 1 h at room temperature the next day after being washed with Tris‐buffered saline with Tween‐20 (TBST, 0.1% Tween‐20). Finally, the membranes were washed with TBST before being immersed in ECL detection reagent (Absin). Subsequently, the imaging system detected signals and captured images (Guangzhou Ewellbio, Tanon‐4600). Blots were analyzed and quantitated with ImageJ software.

### 4.15 RNA‐Sequencing (RNA‐Seq)

Hippocampal tissues from mice in the control group, model group, and NAP2 group were prepared for RNA‐seq. TRIzol reagent (Invitrogen) was used to extract total RNA. Its purity was determined by a Nanophotometer spectrophotometer (IMPLEN), and its integrity was assessed using the Bioanalyzer 2100 system (Agilent Technologies). RNA sequencing was performed using an Illumina HiSeq 2500/4000 instrument (Gene Denovo Biotechnology Co. Ltd.).

### 4.16 Gene Ontology Enrichment Analysis

Gene Ontology (GO) is a globally recognized standard for classifying gene functions in a systematic manner [57]. The GO database includes biological process (BP), cellular component (CC), and molecular function (MF) terms. GO enrichment analysis revealed the functions and components that the identified differentially expressed genes were enriched in. The FPKM values of each differentially expressed gene were averaged and then normalized by the R package (Mfuzz). The fuzzy c‐means algorithm was used for soft clustering to obtain different gene expression trend graphs. The R package cluster profiler (4.0) was used for GO enrichment analysis of genes with different expression trends, and Benjamini–Hochberg was used for *p* value correction (*p*‐adjust).

### 4.17 Statistical Analysis

All quantitative data are presented as the mean ± SEM. Statistical analyses were performed using GraphPad Prism version 9 (GraphPad Software Inc.). *t*‐test or one‐way ANOVA followed by Tukey test were applied to compare parameters among more than two groups. Repeat measured ANOBA was used in latency time from WMW behavior.

## Author Contributions

Zheng Zhang, Huimin Tan, and Fang Shi contributed equally to this work as the first authors. Li Yan and Rui Liao designed the experiments. Zheng Zhang, Xiaoyan Liu, Huimin Tan, Rui Yang, Yuke Wang, and Li‐an Huang performed all the experiments. Jiajia Dai, Weilong Ding, and Junliang Li collected and analyzed the data. Rui Liao and Huimin Tan wrote the manuscript. Xinke Xu and Cheng Chen reviewed and edited the manuscript. All authors have read and approved the final manuscript.

## Funding

This study was funded by the National Natural Science Foundation of China (Grant 82102690), the Basic and Applied Basic Research Foundation of Guangdong Province (Grant 2026A1515011575), the Guangdong Provincial Medical Science and Technology Research Fund (Grant 20211128182437730), the 2024 Joint Project between City, University (College) and Enterprise (Grant 2024A03J0902), the Fundamental Research Funds for the Central Universities (Grant 21626406), and the Horizontal Research Project from the Guangdong Provincial Health Economics Association (Grant 2025‐WJHX‐31).

## Ethics Statement

All experiments were reviewed and approved by the Animal Research Committee of the School of Medicine in Jinan University (approval No.: IACUC‐20180120‐05).

## Conflicts of Interest

The authors declare no conflicts of interest.

## Supporting information


**Figure S1:** NAP2 treatment does not significantly alter hippocampal and cortical morphology or neuronal survival in APP/PS1 mice.
(A, B) Representative H&E staining images showing the histological morphology of the hippocampus (A) and cortex (B) in wild‐type mice, vehicle‐treated APP/PS1 mice, and NAP2‐treated APP/PS1 mice (2/3 mg/kg and 2 mg/kg).
(C) Quantitative analysis of the relative thickness of the cell layer across the different experimental groups.
(D) Quantitative analysis of the relative number of surviving neurons across the different groups. ns indicates no significant difference.
**Figure S2:** Differential gene expression and functional analysis in WT, APP/PS1, and NAP2‐treated APP/PS1 mice.
(A) Heatmap showing the differential gene expression patterns in the three mouse models. The color gradient from blue to red represents the level of gene expression, with red indicating higher expression. The trend of differential gene expression in 16 gene clusters from in WT, APP/PS1(Vehicle), and APP/PS1(NAP2) mice. The patterns in the left heatmap and right trend plot correspond to each other, with each row representing one of the 16 gene clusters.
(B) Gene Ontology (GO) enrichment analysis of the genes in the 16 clusters. Different tables represent the enriched pathways corresponding to the clusters in panel B, with some clusters showing no significant enrichment.

## Data Availability

Data available on request from the authors.
