# Supplementary material for: A Novel Nogo‐A Receptor Antagonist Peptide Impedes Alzheimer's Disease Pathology by Nogo‐A/NgR/ROCK Signaling Pathway
Source: CNS Neurosci Ther. 2026 Aug 13;32(8):e71052. doi: 10.1002/cns.71052 (PMC13473841; doi:10.1002/cns.71052)
Supplement: Supplementary file 1 [file CNS-32-e71052-s001.pdf]

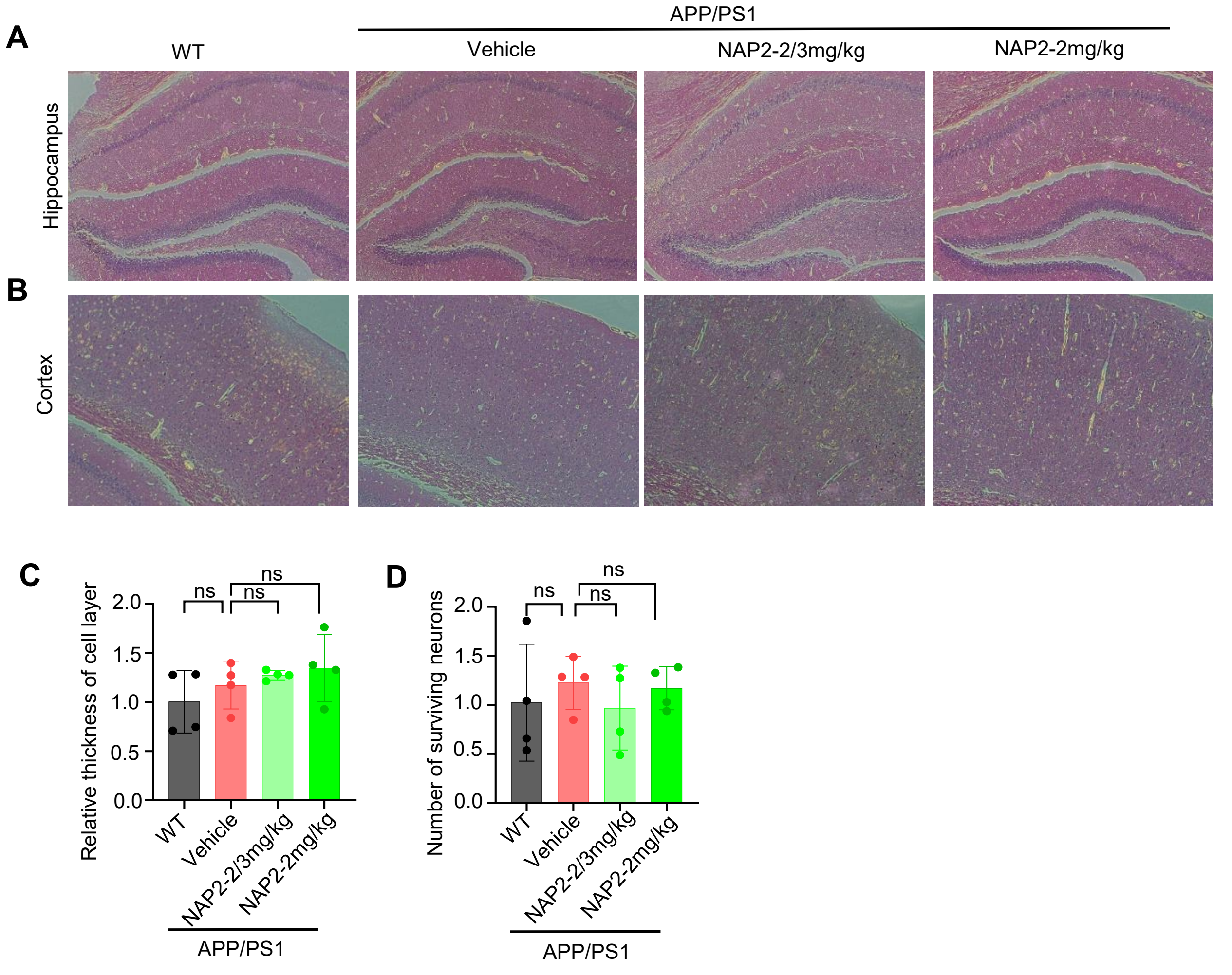

Supplementary Figure 1

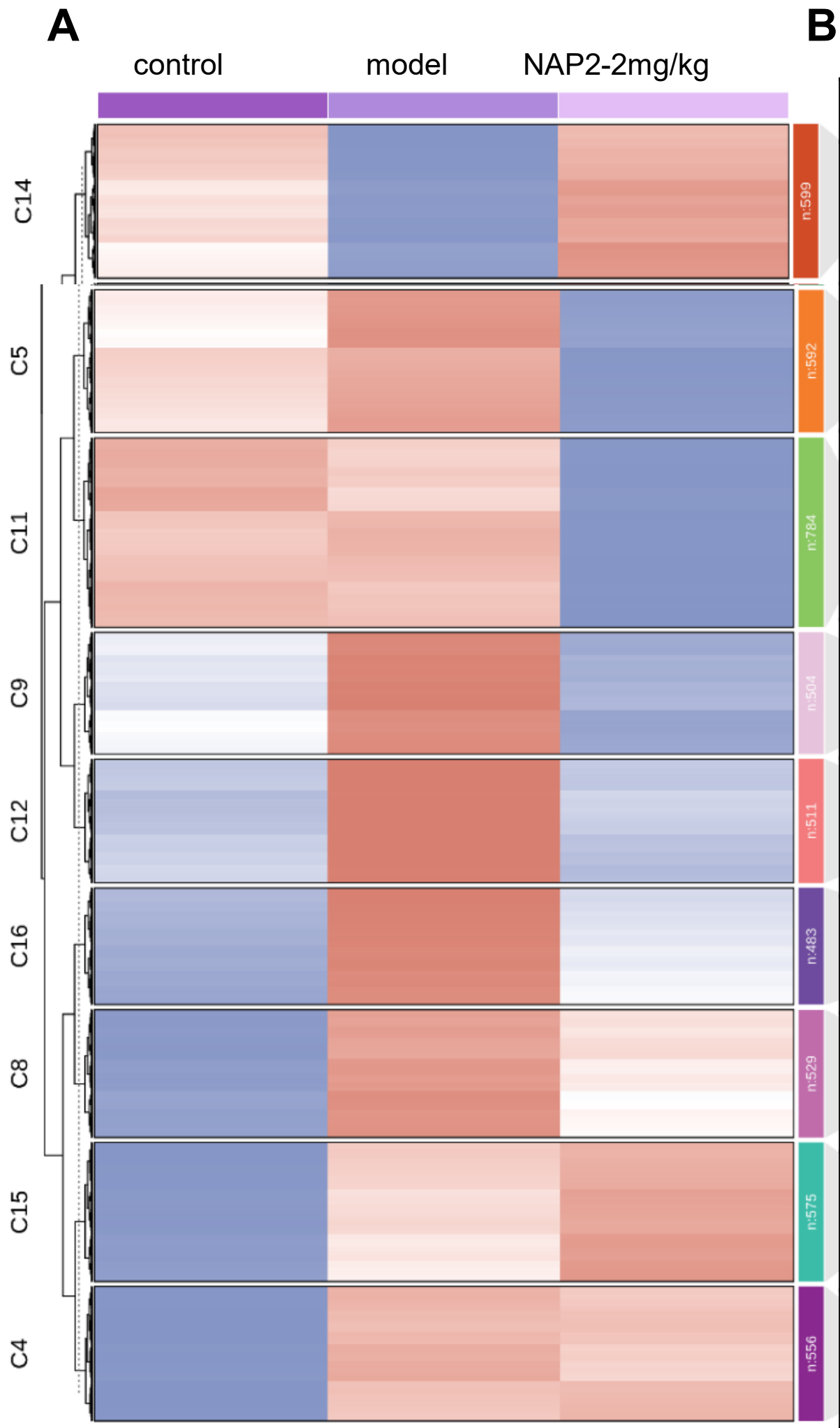

B

Cluster	GO PATHWAY
Gene Size: 599	**Autophagy**<br>**Process utilizing autophagic mechanism**<br>**Cellular component disassembly**<br>**Synapse organization**<br>**Regulation of protein stability**
Gene Size: 592	Ribonucleoprotein complex biogenesis<br>ncRNA metabolic process<br>ncRNA processing<br>Ribosome biogenesis<br>Ribonucleoprotein complex assembly
Gene Size: 784	RNA splicing<br>mRNA processing<br>RNA splicing, via transesterification reactions with bulged adenosine as nucleophile<br>mRNA splicing, via spliceosome
Gene Size: 504	**Mitochondrial membrane organization**<br>**Chromatin organization**<br>**mRNA processing**<br>**Nucleobase-containing compound catabolic prosess**<br>**RNA splicing**
Gene Size: 511	**Axonogenesis**<br>**Cytoplasmic translation**<br>**rRNA processing**<br>**ncRNA processing**<br>**rRNA metabolic process**
Gene Size: 483	**Synapse organization ; Gliogenesis**<br>**Regulation of neurotransmitter levels**<br>**Regulation of cell morphogenesis**<br>**Neurotransmitter transport**
Gene Size: 529	Dendrite development<br>Axonogenesis<br>Regulation of supramoleucular fiber organization<br>Regulation of protein secretion<br>Protein secretion
Gene Size: 575	Cell-substrate adhesion<br>Cell-matrix adhesion<br>Transmembrane receptor protein serine/threonine kinase signaling pathway<br>Cell junction assembly<br>axonogenesis
Gene Size: 556	Dendrite development<br>Dendritic spine development<br>Regulation of synapse structure or activity<br>Synapse organization<br>Regulation of synapse organization

Supplementary Figure 2
